# Contraction frequency and acidemia at birth: A case–control study

**DOI:** 10.1111/aogs.70181

**Published:** 2026-03-04

**Authors:** Johanna Wagner Bjurström, Gisela Rickle, Maria Fogelberg, Frida Ekengård, Andreas Herbst

**Affiliations:** ^1^ Department of Clinical Sciences Lund University Lund Sweden; ^2^ Department of Obstetrics and Gynecology Holbæk Hospital Holbæk Denmark; ^3^ Department of Clinical Medicine, Faculty of Health and Medical Sciences University of Copenhagen Copenhagen Denmark; ^4^ Department of Obstetrics and Gynecology Skåne University Hospital Malmö Sweden; ^5^ Department of Clinical Sciences Lund University Malmö Sweden

**Keywords:** asphyxia neonatorum, cardiotocography, fetal acidemia, tachysystole, uterine contraction, uterine monitoring

## Abstract

**Introduction:**

Consensus is lacking regarding the upper limit for a safe contraction frequency during labor. We aimed to assess the association between different contraction frequencies and acidemia at birth, and whether an association was affected by oxytocin augmentation, concomitant cardiotocography (CTG) classification, and stage of labor.

**Material and Methods:**

This is a case–control study based on CTG traces from births in southern Sweden during 2012–2017. Cases (*N* = 364) had umbilical artery pH <7.1 if born by first‐stage cesarean section and pH <7.05 if born vaginally or by second‐stage cesarean. Controls were included with a 1:2 ratio (*N* = 728) and had pH ≥7.15. CTG traces from the hour prior to birth were assessed blinded to the clinical outcome. Logistic regression was used to estimate odds ratios (OR) with 95% confidence intervals (CI).

**Results:**

After excluding insufficient topography traces, 328 cases and 677 controls remained for analysis. Contraction frequencies 5, 5–6, and ≥6 per 10 min occurred in 15.2%, 19.5%, and 29.6% of cases and in 15.1%, 16.5%, and 11.1% of controls, respectively. The OR (95% CI) for acidemia were 1.6 (1.1–2.4), 1.9 (1.3–2.7), and 4.3 (3.0–6.2), respectively, compared with a reference group of <5. When comparing two separate binary cut‐offs of *>5* and *≥5* contractions, similar strengths of association with acidemia were demonstrated with OR 2.7 (2.1–3.6) and 2.5 (1.9–3.3), respectively. The effect of contraction frequency >5 was similar in spontaneous (*n* = 586) and oxytocin‐augmented (*n* = 419) labor (OR 2.5, 1.7–3.8 vs. OR 2.4, 1.6–3.6), and whether the CTG was normal, suspicious, or pathological according to the Swedish CTG template (OR 2.1, 1.3–3.3; OR 3.5, 1.6–7.6; and OR 2.6, 1.4–4.7, respectively). The study lacked power to study any association during the first stage of labor.

**Conclusions:**

Increasing contraction frequency is incrementally associated with fetal acidemia. Although an increased risk of acidosis is evident at contraction frequencies as low as five, such frequencies are common while acidemia is uncommon, implying a poor positive predictive value. A contraction frequency of more than five is associated with acidemia in both spontaneous and oxytocin‐augmented labor, and even when the fetal heart trace is normal.

## Graphical abstract

This case–control study shows an association by exposure level between contraction frequency during the final hour before birth and fetal acidemia. Effect sizes are similar regardless of oxytocin use. Contraction frequencies above both the conventional and revised thresholds are common, indicating poor positive predictive value.

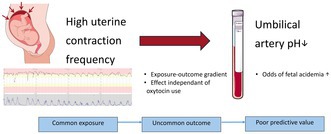

**Abbreviations**
ACOG: American college of obstetricians and gynecologists
CF: contraction frequency
CI: confidence interval
CTG: cardiotocography
FIGO: International federation of gynecology and obstetrics
IUPC: intrauterine pressure catheter
Min: minutes
NICE: National institute of clinical excellence
OR: odds ratio
TS: tachysystole

**Key Message**

This study demonstrates an association between contraction frequency prior to birth and fetal acidemia, following a dose–response pattern. The effect size is similar with/without oxytocin. High contraction frequencies are, unlike acidemia, common—implying a poor positive predictive value.

## 1 INTRODUCTION

While uterine contractions are the primary mechanical force responsible for the initiation and progression of labor,^1^, ^2^ they also compromise the uteroplacental blood circulation, causing intermittently decreased oxygen supply to the fetus.^3^

It is well established that if the contractions are too frequent, *tachysystole* (TS), the fetus will be exposed to insufficient restitution and gradually decreasing oxygen levels which may lead to progressive metabolic acidosis at birth. Previous studies measuring changes in fetal oxygen saturation during oxytocin‐augmented contractions have attempted to find the threshold for a safe average contraction frequency (CF). Two separate prospective studies indicated that contractions more often than every two minutes will lead to a gradual deterioration in fetal oxygen saturation,^4^, ^5^ whereas a third retrospective study demonstrated gradually deteriorating fetal saturation with a contraction frequency of five or more contractions per 10 min.^6^ The most common threshold for CF stated in fetal monitoring guidelines has traditionally been *no more than five* contractions in 10 min.^7^, ^8^, ^9^ Recently, Evans et al. challenged this threshold,^10^ and NICE subsequently updated their cardiotocography (CTG) guidelines accordingly. According to the updated NICE guidelines from 2022, five contractions per 10 min is viewed as an intrapartum risk factor indicating intervention to lower the CF, regardless if the contractions are augmented or not.^11^

Previous studies have addressed the association between CF and different fetal outcome variables. These studies are heterogeneous when it comes to outcome measure, definition of TS, and for how long and when during labor the contractions are studied. They also rarely consider the concurrent FHR trace, or if the contractions are spontaneous or augmented with oxytocin. The systematic review by Reynolds et al. therefore concluded that evidence of an association between CF and poor short‐term or long‐term neonatal outcome is inconsistent and insufficient.^12^

The objective of this case–control study is to investigate if CF and/or the presence of TS during the last 60 min prior to birth is associated with fetal acidemia at birth. Subanalyses are stratified by contraction type (spontaneous vs. oxytocin‐augmented), stage of labor (first vs. second), and fetal heart rate classification (normal, suspicious, or pathological).

## 2 MATERIAL AND METHODS

This was a case–control study. We used the CTG traces from births at Skåne University Hospital and Helsingborg Hospital during 2012–2017, compiled in two previous studies,^13^, ^14^ and subsequently complemented.^15^ Inclusion criteria for both cases and controls were singleton pregnancy, labor after ≥34 + 0 gestational weeks, and available CTG traces for ≥30 min prior to birth. A maximum gap of 30 min from the end of the CTG trace to birth was allowed for cesarean births.

Cases (*N* = 364) were defined as having acidemia at birth as an indicator for exposure to hypoxia during labor. If birth was given vaginally or by second‐stage cesarean section, acidemia was defined as cord arterial or venous pH <7.05. If birth was given by first‐stage cesarean section, acidemia was defined as pH <7.10. The reasoning behind different pH cut‐offs was that the fetal pH decreases during the second stage of labor.^16^, ^17^ The controls were included with a ratio of 1:2 (*N* = 728) as the consecutive two neonates born after each case at the same hospital matching the inclusion criteria and with umbilical cord arterial and venous pH ≥7.15 and at least 0.02 apart, and Apgar scores ≥9 at 5 and 10 min after birth. The two controls to every case were matched to the same stage of labor; the controls for the cases born either vaginally or by second‐stage cesarean section had CTG traces included for analysis from the last 30–60 min prior to vaginal birth. The controls for cases born by first‐stage cesarean section could have different modes of birth, but their CTG traces included for analysis were from the same cervical dilatation as the corresponding cases had been prior to cesarean birth. The cases and controls were thereby matched on stage of labor based on cervical dilatation, hospital, and approximate date and time during the study period.

The 1092 tocography traces of the last 60 min were assessed by the corresponding author (JWB), blinded to clinical outcome. The CF during the last 30 min, as well as the second last 30 min of the traces, was determined. The fractions of the tocography traces deemed not assessable for contractions were noted. We excluded CTGs with tocography traces allowing reliable assessment of the CF for less than 20 min during both 30‐min periods.

CF was registered as average number of contractions per 10 min during each of the two 30‐min periods. The CF of the CTG traces was determined by the highest frequency demonstrated during any of the two periods. When studying different cut‐offs for CF and their association with acidosis, the data were stratified in three different ways (Table 2). First, the data were stratified into four groups to explore a potential dose–response relationship, with contraction frequencies defined as follows: “less than five” (<4.75), “five” (4.76–5.25), “between five and six” (5.26–5.75), and “six or more” (≥5.76 contractions/10 min). The second and third stratifications were binary to enable comparison of the conventional and revised thresholds, defined as “more than five” (>5.00 contractions/10 min) and “five or more” (≥5.00 contractions/10 min), respectively. Furthermore, JWB and an additional author (MF) assessed whether the CTG traces fulfilled the criteria for tachysystole as defined by FIGO (more than five contractions per 10 min, on average seen over a 30‐min period, or more than five contractions per 10 min in two subsequent 10 min periods).^8^ If the two assessors disagreed upon whether the trace displayed tachysystole or not, a third author (AH) assessed the trace and the assessment by majority was used for analysis.

The fetal heart rate characteristics of each CTG trace had previously been systematically assessed by two of the authors. The features assessed were the baseline fetal heart rate, heart rate variability, decelerations, accelerations, and contractions. If they did not agree, a third assessor would evaluate the trace, and the majority ruling was applied.^18^ The assessments were compiled to classify the trace according to the FIGO CTG interpretation guideline from 2015, and to the CTG template used in Sweden since 2017 (SWE17), both of which are divided in three tiers: normal, suspicious, and pathological. Neither includes CF in the classification. Electronic patient charts were reviewed to determine if the contractions were augmented by oxytocin or if misoprostol had been administered <6 h prior to birth.

This study used previously obtained data.^13^, ^14^ Therefore, no power analysis was performed prior to conducting the study. SPSS Statistics version 29.0 (IBM Corp., Armonk, NY) was used for data analysis. Baseline characteristics were presented by mean and standard deviation if normally distributed, and by median and interquartile range if not normally distributed. For comparison of the mean CF, a student's *t*‐test was performed. The association between the presence of TS and fetal acidemia was analyzed using binary logistic regression, with OR and 95% CI calculated. CF was either dichotomized or categorized into four ordered groups, and analyzed using binary and ordinal logistic regression, respectively. A *p*‐value <0.05 was considered statistically significant.

## 3 RESULTS

After excluding 36 cases and 51 controls which did not have at least 20 min of assessable tocography traces during any of the two 30‐min periods studied, 328 cases and 677 controls were included in the analyses. In total, 1005 CTG traces were included: 191 from the first stage of labor and 814 from the second stage of labor.

Baseline characteristics are summarized in Table 1. Cases were more likely to be nulliparous, included more women with preeclampsia and pre‐existing or gestational diabetes mellitus, more often received oxytocin, and had meconium‐stained amniotic fluid, fever, or signs of infection.

**TABLE 1:** Baseline characteristics and mode of birth.

	Cases with acidemia, *N* = 328	Controls without acidemia, *N* = 677
BMI, median (IQR)	24.2 (22.1–27.5)	23.5 (21.4–26.7)
Missing, *n* (%)	6 (1.8%)	16 (2.4%)
Nulliparous, *n* (%)	206 (62.8%)	335 (49.5%)
Missing, *n* (%)	0	0
Smoking, *n* (%)	19 (5.8%)	49 (7.2%)
Missing, *n* (%)	5 (1.5%)	10 (1.5%)
Birthweight		
Mean (SD), g	3596 (519)	3554 (467)
< 2.5 kg, *n* (%)	5 (1.5%)	9 (1.3%)
>4.5 kg, *n* (%)	10 (3.0%)	17 (2.5%)
Missing, *n* (%)	3 (0.9%)	0 (0%)
Gestational weeks^days^, mean (SD)	40^2^ (1^2^)	40^0^ (1^2^)
Missing, *n*	0	0
Oxytocin infusion, *n* (%)	183 (55.8%)	236 (34.9%)
Missing, *n*	0	0
Misoprostol within 6 h, *n* (%)	15 (4.6%)	3.7 (5.5%)
Missing, *n*	0	0
Preeclampsia, *n* (%)	12 (3.7%)	16 (2.4%)
Missing, *n*	0	0
Pregestational or gestational diabetes mellitus, *n* (%)	15 (4.6%)	20 (3.0%)
Missing, *n* (%)	0	0
Meconium‐stained fluid, *n* (%)	88 (26.8%)	127 (18.8%)
Missing, *n* (%)	1 (0.3%)	0 (0%)
Fever/signs of infection, *n* (%)	11 (3.4%)	7 (1.0%)
Missing, *n*	0	0
Mode of birth, *n* (%)		
Non‐assisted vaginal birth	166 (50.6%)	628 (92.8%)
Vacuum extraction/forceps	79 (24.1%)	38 (5.6%)
Cesarean section	83 (25.3%)	11 (1.6%)
Missing, *n*	0	0

The mean average CF during the period studied was 4.9 (SD 1.1) for cases, vs. 4.3 (SD 1.0) for controls (*p* < 0.001). Tachysystole, as defined by FIGO, was present in 46.6% of the cases vs. 25.8% of the controls (*p* < 0.001). A gradient in effect size was observed, with the OR increasing incrementally from 1.6 to 4.3 with higher CF (Table 2). When analyzing CF binarily with two different cut‐offs, *more than five* contractions per 10 min was associated with acidemia (OR 2.7, 95% CI 2.1–3.6) compared with a reference group of *five or less*, as was *five or more* (OR 2.5, 95% CI 1.9–3.3) when compared to a reference group of *less than five*. In the subgroup analyses (Tables 3 and 4), we used a single cut‐off of *more than five* contractions per 10 min. Cases and controls more frequently had a CF above this cut‐off if oxytocin was administered (cases 56.8% with oxytocin vs. 44.8% without, controls 35.2% with oxytocin vs. 24.3% without); however, the strength of the association with acidemia was similar with and without oxytocin (OR 2.4, 95% CI 1.6–3.6 vs. OR 2.5, 95% CI 1.7–3.8, respectively). The association between *more than five* contractions per 10 min and acidemia was significant for the second stage of labor, but not for the first (Table 3).

**TABLE 2:** Associations between different contraction frequencies and tachysystole, with acidemia at birth.

	Cases (*N* = 328)	Controls (*N* = 677)	OR (95% CI)	*p*‐value
Average contraction frequency per 10 min, mean (SD)	4.9 (1.1)	4.3 (1.0)		<0.001
Tachysystole^a^				
No	175 (53.4%)	502 (74.2%)	Ref.	
Yes	153 (46.6%)	175 (25.8%)	2.5 (1.9–3.3)	<0.001
Highest average contraction frequency per 10 min, for ≥30 min during the last hour prior to birth^b^				
Less than five	117 (35.7%)	388 (57.3%)	Ref.	
Five	50 (15.2%)	102 (15.1%)	1.6 (1.1–2.4)	0.016
Between five and six	64 (19.5%)	112 (16.5%)	1.9 (1.3–2.7)	<0.001
Six or more	97 (29.6%)	75 (11.1%)	4.3 (3.0–6.2)	<0.001
Five or less	159 (48.5%)	487 (71.9%)	Ref.	
More than five	169 (51.5%)	190 (28.1%)	2.7 (2.1–3.6)	<0.001
Less than five	122 (37.2%)	405 (59.8%)	Ref.	
Five or more	206 (62.8%)	272 (40.2%)	2.5 (1.9–3.3)	<0.001

^a^ As defined by FIGO: the occurrence of more than five contractions in 10 min, in two successive 10‐min periods, or averaged over a 30‐min period.

^b^ The first stratification of contraction frequency groups was defined as “less than five” = ≤4.75, “five” = 4.76–5.25, “between five and six” = 5.26–5.75, “six or more” = ≥5.76. The second and third binary stratifications were defined as “more than five” = >5.00 and “five or more” = ≥5.00.

**TABLE 3:** Associations between contraction frequency >5/10 min with acidemia at birth in four subgroups; (1) no oxytocin augmentation, (2) oxytocin augmentation, (3) first stage of labor and (4) second stage of labor.

	Cases, *N* = 328	Controls, *N* = 677	OR (95% CI)	*p*‐value
No oxytocin	145	441		
≤5 per 10 min	80 (55.2%)	334 (75.7%)	Ref.	
>5 contractions per 10 min^a^	65 (44.8%)	107 (24.3%)	2.5 (1.7–3.8)	<0.001
Oxytocin augmented	183	236		
≤5 per 10 min	79 (43.2%)	153 (64.8%)	Ref.	
>5 contractions per 10 min^a^	104 (56.8%)	83 (35.2%)	2.4 (1.6–3.6)	<0.001
1st stage of labor	62	129		
≤5 per 10 min	51 (82.3%)	114 (88.4%)	Ref.	
>5 contractions per 10 min^a^	11 (17.7%)	15 (11.6%)	1.6 (0.7–3.8)	0.25
2nd stage of labor	266	548		
≤5 per 10 min	108 (40.6%)	373 (68.1%)	Ref.	
>5 contractions per 10 min^a^	158 (59.4%)	175 (31.9%)	3.1 (2.3–4.2)	<0.001

^a^ During at least 30 min of the period studied (≤60 min).

**TABLE 4:** Associations between contraction frequency >5/10 min with acidemia at birth in six subgroups; normal, suspicious, or pathological fetal heart trace according to FIGO 2015 and SWE17, respectively.

	Cases, *N* = 328	Controls, *N* = 677	OR (95% CI)	*p*‐value
Normal fetal heart rate (FIGO 2015)	23	241		
≤5 per 10 min	16 (69.6%)	190 (78.8%)	Ref.	
>5 contractions per 10 min^a^	7 (30.4%)	51 (21.2%)	1.6 (0.6–4.2)	0.309
Suspicious fetal heart rate (FIGO 2015)	135	383		
≤5 per 10 min	65 (48.1%)	265 (69.2%)	Ref.	
>5 contractions per 10 min^a^	70 (59.9%)	118 (30.8%)	2.4 (1.6–3.6)	<0.001
Pathological fetal heart rate (FIGO 2015)	170	53		
≤5 per 10 min	78 (45.9%)	32 (60.4%)	Ref.	
>5 contractions per 10 min^a^	92 (54.1%)	21 (39.6%)	1.8 (1.0–3.4)	0.07
Normal fetal heart rate (SWE‐17)	87	547		
≤5 per 10 min	48 (55.2%)	393 (71.8%)	Ref.	
>5 contractions per 10 min^a^	39 (44.8%)	154 (28.2%)	2.1 (1.3–3.3)	0.002
Suspicious fetal heart rate (SWE‐17)	50	67		
≤5 per 10 min	24 (48%)	51 (76.1%)	Ref.	
>5 contractions per 10 min^a^	26 (52%)	16 (23.9%)	3.5 (1.6–7.6)	0.002
Pathological fetal heart rate (SWE‐17)	191	63		
≤5 per 10 min	87 (45.5%)	43 (68.3%)	Ref.	
>5 contractions per 10 min^a^	104 (54.5%)	20 (31.7%)	2.6 (1.4–4.7)	0.002

^a^ During at least 30 min of the period studied (≤60 min).

Regardless of the concomitant heart trace being normal, suspicious or pathological according to SWE17; a CF of *more than five* per 10 min was significantly associated with acidemia: OR 2.1, 95% CI 1.3–3.3; OR 3.5, 95% CI 1.6–7.6; and OR 2.6, 95% CI 1.4–4.7, respectively (Table 3). When classifying the fetal heart traces according to FIGO, a significant association between a CF of more than five and acidemia was only present if the fetal heart rate was classified as suspicious (OR 2.4, 95% CI 1.6–3.6).

## 4 DISCUSSION

In this case–control study, an increasing CF was incrementally associated with fetal acidemia, with OR ranging from 1.6 at five contractions per 10 min to 4.3 at six or more contractions per 10 min. The association was similar for spontaneous and oxytocin‐augmented contractions.

A strength of this study is the large number of neonates with acidemia at birth included. Participants were sampled from a population where umbilical blood sampling is routinely used, avoiding selection bias. Another strength of the study is that assessments of CTG traces were blinded to the outcomes. Limitations of this study include the small number of cases with acidemia in the first stage of labor and that the study sample was too small for subanalyses of the different CTG classification groups according to the FIGO template. In addition, 10% of cases and 7% of controls were excluded due to insufficient tocography quality. An important limitation concerns the comparability of control CTG traces. For vaginal and second‐stage cesarean births, controls were matched by hospital, approximate time, and stage of labor, with CTGs sampled from the hour preceding birth. For first‐stage cesarean deliveries, however, control CTGs were sampled at the same cervical dilation as the case rather than immediately prior to birth. This strategy was chosen to enable comparison within the same stage of labor, as contraction patterns differ substantially between labor stages. Although this approach improves stage‐specific comparability, it cannot fully account for potential changes in uterine activity or fetal condition later in labor among controls. However, as controls were subsequently born without acidemia and umbilical pH normally decreases during labor, it is unlikely—though it cannot be completely excluded—that the sampled control CTG represented an acidemic fetus at the time of recording. Identifying an optimal control group is likely not possible, and we believe that the chosen strategy represents a pragmatic and clinically relevant approach under these circumstances.

An association between CF and acidemia fulfills several of Bradford Hills criteria for causation: temporality, biological gradient, plausibility, consistency from previous studies and coherence from animal studies. Still, this observational study can only show an association between exposure and outcome, and potential confounders are important to reflect on. Confounders might include induction of labor, oxytocin augmentation, placental abruption, chorioamnionitis and prolonged second stage of labor and bearing down. We considered oxytocin augmentation to be the most likely confounding factor, which is prevalent enough to affect an association substantially. We therefore performed subanalyses of the association between CF and acidemia for women with and without oxytocin‐augmented labor. We also acknowledge the potential for reverse causation, as abnormal fetal heart rate patterns may have influenced subsequent management with tocolytics and/or oxytocin. Since we found a similar association between a high CF and acidemia in spontaneous and oxytocin‐augmented labor, and a similar association regardless of the classification of the fetal heart rate trace, substantial confounding of clinical importance appears unlikely. Since this is a retrospective study of labors where interventions had been undertaken in cases of suspected fetal distress, it is possible that associations between frequent contractions and acidemia may have been weaker than if no interventions had been allowed. From an ethical perspective, such a study would have been unacceptable.

The largest systematic review on the topic did not find a consistent association between increased uterine activity and adverse neonatal outcome or proxy neonatal measures, such as umbilical artery pH.^12^ However, our findings are in line with several previous observational studies,^10^, ^19^, ^20^, ^21^ which, like ours, have focused on the time period immediately before birth—the period where CTG characteristics have previously been found to be predictive to neonatal outcome.^22^, ^23^

Our present study focused on CF as the exposure, whether the tocography trace was recorded externally or internally. Uterine contractile activity can be quantified by frequency, duration, relaxation time, amplitude, baseline tone, and Montevideo units (MVU).^1^ External tocometry can quantify the CF and may also display duration of contractions and the relaxation time if the signal quality is good. Measurement of amplitude, baseline tone, and MVU requires an intrauterine pressure catheter (IUPC).^1^, ^2^, ^19^ Most studies on uterine activity and different neonatal outcome measures quantify and analyze CF alone, as IUPCs are not routinely used. Two of the studies that only included patients with internal contraction registrations may be subject to considerable selection bias, as their study populations consisted of patients in whom IUPCs were placed for clinical indications. One of them found that the uterine contraction parameters supplied by an IUPC were not more strongly correlated with fetal pH than contraction frequency alone.^19^ The second study found that tachysystole was associated with adverse neonatal outcome; however, contraction duration, relaxation time, MVU, and baseline tone were not.^24^ A third study which included patients that had been randomized to an IUPC in a previous study found that neither CF, MVU, nor baseline tone were associated with umbilical artery pH ≤7.10.^25^

We observed a dose–response pattern with progressively stronger effect sizes with higher CF levels. A cohort study from 2022 reported similar findings, suggesting to potentially detect fetal compromise earlier by lowering the threshold for safe uterine activity from no more than five to no more than four contractions per 10 min as averaged over 30 min.^10^ Our results indicate that the risk of acidemia was only modestly increased for the group of CTG traces with precisely five contractions per 10 min (OR 1.6; 95% CI 1.1–2.4). CF above the conventional versus above the revised threshold displayed similar effect sizes—with OR of 2.7 (95% CI 2.1–3.6) and OR of 2.5 (95% CI 1.9–3.3), respectively. However, it is worth noting that with the lower threshold, as many as 40% of CTG traces of non‐acidemic fetuses would be considered to have too frequent contractions, which may constitute a problem in clinical management.

We were interested in whether an elevated CF imposed any risk of acidemia in the presence of a normal heart rate pattern. The results indicated that a high CF was associated with acidemia in the presence of normal as well as suspicious and pathological heart rate patterns, when classifying the traces according to SWE17, although obviously the absolute risk of acidemia is lower with normal heart rate patterns. With the FIGO classification, a significant association between CF and acidemia was only found when the heart rate pattern was classified as suspicious. The number of acidemic fetuses with normal heart rate patterns according to FIGO, as well as the number of non‐acidemic fetuses with pathological heart rate patterns, may have been too small to adequately analyze these subgroups. High CF during the first stage of labor was also too rare to allow analysis of any possible association.

The role of CF in different CTG interpretation templates varies. In the NICE guidelines for fetal monitoring, CF is viewed as a possible underlying cause of fetal heart rate abnormalities. A CF of more than four per 10 min is an “amber feature” and results in a suspicious CTG. If the feature occurs with another amber feature, the CTG is classified as pathological. By contrast, CF is not considered when classifying a CTG trace according to the current Swedish CTG template, FIGO or ACOG.^7^, ^8^, ^9^, ^11^ Our results, demonstrating a CF of more than five as having an independent association with acidemia even with a normal CTG according to the Swedish CTG template, suggest that CF should be included in classification templates. The risk factor of frequent contractions is often iatrogenic^20^ and can be modified by labor management. Attention should therefore be paid to the monitoring of not only fetal heart rate but also the tocography trace, and high CF should be avoided. Including a CF of more than five or tachysystole as a “suspicious” criterion in a three‐tier CTG template would underscore CF as a risk factor. However, since CF as a single CTG characteristic yields no more than moderate effect sizes in comparison with aberrant fetal heart rate patterns,^18^ it may not on its own be enough to indicate other interventions than halting or reducing an ongoing oxytocin infusion. With a prevalence of acidemia (as defined in this study) of 1.3% in our background population, the results of the present study would imply a positive predictive value of only 2.4% (95% CI 2.0–2.8) for a CF of more than five. We suggest that in case of a CF exceeding the set threshold in non‐oxytocin‐augmented labor, expectant management with close continuous monitoring may be sufficient if the fetal heart rate pattern is normal. If the pattern changes to suspicious or pathological, further interventions should be considered.

## 5 CONCLUSION

A high CF during the last 60 min prior to birth is associated with fetal acidemia in a dose‐dependent manner. An association is seen even when contractions are spontaneous, and regardless of whether the concomitant fetal heart rate trace is normal, suspicious, or pathological according to the CTG template currently used in our institutions (SWE17). The upper limit for normal CF varies between four and five contractions per 10 min in different international guidelines. Both cut‐offs are challenged by the high prevalence of CF above threshold in CTG traces of non‐acidemic fetuses.

## AUTHOR CONTRIBUTIONS

**Johanna Wagner Bjurström:** Conceptualization; funding application; CTG assessment; data analysis; statistical analyses; writing first manuscript draft; correspondence. **Gisela Rickle:** CTG assessment; manuscript revision. **Frida Ekengård:** Funding application; data retrieval; CTG assessment; manuscript revision. **Maria Fogelberg:** CTG assessment; manuscript revision. **Andreas Herbst:** Conceptualization; funding application; CTG assessment; data analysis; manuscript revision.

## FUNDING INFORMATION

Grants from Skane University Hospital Funds and Foundations (organization number 232100‐0255), and Doctor Margaretha Nilssons Foundation (organization number 802480–5718) financed this study.

## CONFLICT OF INTEREST STATEMENT

None of the authors have any conflict of interest to declare.

## ETHICS STATEMENT

Ethical approval was obtained from the Regional Ethical Review Board in Lund (Dnr 2016/371) on May 24, 2016. Informed consent from the patients was not demanded for this retrospective study of cardiotocographic patterns.

## Data Availability

The data that support the findings of this study are available from the corresponding author upon reasonable request.
